# Simulant molten core–concrete interaction experiments in view of understanding Fukushima Daiichi Nuclear Power Station Cs-bearing particles generation mechanism

**DOI:** 10.1038/s41598-024-56972-9

**Published:** 2024-03-19

**Authors:** Hugo Laffolley, Christophe Journeau, Bernd Grambow

**Affiliations:** 1 CEA, DES, IRESNE, DTN, Severe Accident Experimental Laboratory, Cadarache, 13108 St-Paul-lez-Durance, France; 2 SUBATECH (IMT Atlantique, CNRS-IN2P3, University of Nantes), 44307 Nantes, France

**Keywords:** CsMP, Fukushima Daiichi, Radioactive aerosols, Environmental sciences, Materials science, Nuclear energy, Environmental sciences, Materials science, Nuclear energy

## Abstract

The Fukushima Daiichi accident resulted in the release of a novel form of radioactive Cs contamination into the environment, called Cs-bearing microparticles (CsMP). CsMPs constitute a substantial portion of the radioactive pollution near the nuclear power station and traveled beyond several hundred kilometers. Extensive characterization of the CsMPs revealed an amorphous silica matrix, along with Cs and other minor or trace elements such as Fe and Zn. This study explores the unclear generation mechanism of CsMPs by conducting experimental molten core concrete interactions (MCCI) as a source of Si and analyzing the resultant aerosols. The findings demonstrate that MCCI is in capacity to produce spherical submicronic and micronic particles, primarily composed of amorphous silica and incorporating elements akin to CsMPs. A humid atmosphere is found to favour an even closer chemical composition. Examination of the internal structure of the synthesized particles unveils pores and numerous crystalline nanoinclusions possibly serving as nucleation sites for CsMP formation through the condensation of Si-rich vapors.

## Introduction

On March 11, 2011, the Fukushima Daiichi Nuclear Power Station (1F) experienced three reactor core meltdowns, catastrophic events caused by the loss of power following the partial flooding of the 1F site^1^. This incident, ranked the highest level on the International Nuclear Event Scale, resulted in the release of radioactive materials into the environment a few days after the initiating events^2^.

Subsequent airborne monitoring of radiocesium identified a novel form of radioactive particle, named Cs-bearing microparticles (CsMP), the first time at Tsukuba (170 km southwest of 1F) in the night of March 14th to 15th^3^. CsMPs are mostly made of an amorphous silica matrix containing various elements, including radioactive cesium, and are categorized into type-A (micrometric-sized particles, originating from Unit 2 or Unit 3, high specific radioactivity) and type-B (tens to hundreds of micrometers, originating from Unit 1, low specific radioactivity)^4–8^. Type-A CsMPs tend to be spherical particles and could travel on hundreds of kilometers due to their micrometric size^9,10^.

Determining the proportion of Cs radioactivity attributable to CsMPs in a given soil sample proves challenging. Nevertheless, it is proposed that in close proximity to 1F, CsMPs may locally contribute significantly to Cs radioactivity^11,12^. The cesium encapsulated within CsMPs has a limited transferability to the environment due to the low solubility of the silica matrix in water^13–15^. Combining their minute size and low solubility, aspirable CsMPs may cause prolonged radiation doses locally, in cases of deep penetration into an individual’s respiratory system, surpassing the biological half-life of Cs in its ionic form. Moreover, the presence of this new type of Cesium-bearing aerosols will affect the fission product chemistry in the containment while it is not currently modelled in severe accidents such as ASTEC^16^ or MAAP^17^.

Despite extensive characterization of type-A CsMPs in the years following their discovery, a consensus on the detailed mechanism of their generation remains elusive. It is evident that the source must entail a sufficient amount of silicon to form the particles’ matrix, with other elements such as Fe, Zn, or Cs integrated into the particles.

One proposed origin of CsMPs suggests an interaction between Cs (as $\mathrm {Cs_{2}O}$) and up to 1% silicon present in the stainless steel of the internals and the reactor pressure vessel (RPV) at high temperatures. Experimental and thermodynamic evidences support the formation of oxidic phases rich in Fe, Si, and Cs, which could later evolve into type-A CsMPs through remelting^18^. This hypothesis has the benefit of being valid for both in-vessel and ex-vessel phenomena. Another proposed mechanism suggests a gas-solid interaction between CsOH, likely formed in humid atmospheres^19^, and insulation materials such as calcium silicate at high temperatures^20^. Experimental work demonstrated the formation of complex chemical species, such as $\mathrm {CsAlSiO_{4}}$, potentially leading to CsMPs. Additionally, it has been theorized that during the Unit 3 hydrogen explosion, the safety gas treatment system, equipped with high-efficiency particulate air filters, may have atomized to form type-A CsMPs^21,22^. However, these hypotheses lack experimental validation as very similar particles could not be, to our best knowledge, synthesized.

Consequently, a fourth hypothesis, mentioned several times in the literature, suggests that type-A CsMPs may result from molten core-concrete interaction (MCCI) following RPV failure^23,24^. This theory relies on the potential interaction at high temperatures, between the zirconium fraction of sub-oxidized corium and the silica present in the concrete^25^, particularly the basaltic concrete of 1F rich in SiO$_{2}$. This interaction could produce silica-rich gases that condense into micrometric spherical particles.

Preliminary investigations have demonstrated the feasibility of generating microparticles resembling CsMPs in shape and size, along with a comparable chemical composition, through small-scale MCCI experiments (about 20 g of reagents) in the VITI furnace at the CEA Cadarache^26^. Simultaneously, another research team observed similar results while simulating MCCI using a distinctly different experimental setup^27^. Consequently, additional experiments in the VITI furnace, coupled with more in-depth analyses, have been conducted to reinforce the theoretical framework and gain a nuanced understanding of the generation mechanism of type-A particles.

## Results

### Objective

The primary objective of this study is to produce and gather aerosols resulting from MCCI, in conditions approaching those estimated during the accident of 1F, in order to examine their morphology and chemical composition. These particles will be compared with data accumulated over the last decade concerning type-A CsMP, aiming to enhance our understanding of the generation mechanism of type-A CsMP. To achieve this, a small-scale experimental setup has been devised to induce the reaction of a mixture of concrete and prototypic corium, at a temperature about 2000–2200 $^\circ$C, defined by thermodynamic calculations^28^, under atmospheric pressure, and within a controlled atmosphere (pure nitrogen or a mixture of steam and nitrogen). This experiment is based on the fact that at high temperature, Zr (from fuel cladding) is easily oxidised in an exothermic reaction, in particular by the silica contained in the basaltic concrete to form silicon-rich gas, and consequently particles^25^. The aerosols generated are subsequently collected on a filter for post-experimental analyses.

### Size and morphology of the generated aerosols

The aerosols generated during our experiments were subjected to airborne particle mass size distribution analysis using a low-pressure impactor. The geometric diameter was determined based on the aerodynamic diameter, assuming spherical particles with a density similar to that of silica glass of low concentration in alkali species (2.25 kg m$^{-3}$)^29^. The 250 nm cutoff diameter stage collected the maximum mass of aerosols, corresponding to a geometric diameter of 170 nm (Figure S1). In comparison, the mass collected on the 1.6 $\upmu$m and 2.5 $\upmu$m cutoff stages, corresponding to geometric diameters of 1.1 $\upmu$m and 1.7 $\upmu$m respectively, was 15 times lower than that of the stage with the maximum mass.

A comparison of the aforementioned measurements was performed using Scanning Electron Microscopy (SEM) images. To count a large number of particles, the CELLPOSE algorithm, based on artificial intelligence (AI), was employed^30^. Two SEM images taken at a magnification of approximately 18,000 and 10,000 allowed the identification of 354 and 821 regions of interest (ROI) respectively, which correspond to potential submicrometric-sized particles (Figure S2). The median diameter was determined by considering both the maximum Feret diameter and the mean of the major and minor axes of an ellipse that best fits the projected area of each particle without exceeding its boundaries. For the two images, the measured Feret diameters were found to be 208 nm and 211 nm, respectively. The ellipse method diameters were measured to be 165 nm and 166 nm, respectively. These measurements, particularly those related to the ellipse method diameter, are consistent with the size distribution obtained through impaction analysis.

SEM observations on the prepared samples revealed the presence of micrometric-sized particles resembling CsMPs. Their occurrence is significantly lower than that of submicrometric-sized particles, but their size makes them easily distinguishable even at low magnification levels on the SEM. Considering the cascade impactor stages 11 to 14, which have aerodynamic cutoff diameters of 1.6 $\upmu$m, 2.5 $\upmu$m, 3.6 $\upmu$m, and 5.3 $\upmu$m respectively, a rough estimation of the rate of generation of micrometric-sized particles can be achieved. Stage 15, with the highest cutoff diameter, is not considered, as large dust pollution can be collected. For stages 11 to 14, the corresponding geometric cutoff diameters are respectively 1.1 $\upmu$m, 1.7 $\upmu$m, 2.4 $\upmu$m, and 3.5 $\upmu$m, considering a particle material density of 2.25 kg m$^{-3}$ and a spherical shape. By making the hypothesis that every particle collected on each stage has a diameter equal to the geometric cutoff diameter, and considering the respective mass gain of these stages (Figure S1) during the 25 s collection time, the number of micrometric-sized particles collected on stages 11 to 14 can be estimated to be around $2\times 10^{8}$ particles or $9 \times 10^{6}$ particles.s$^{-1}$.

**Figure 1:**
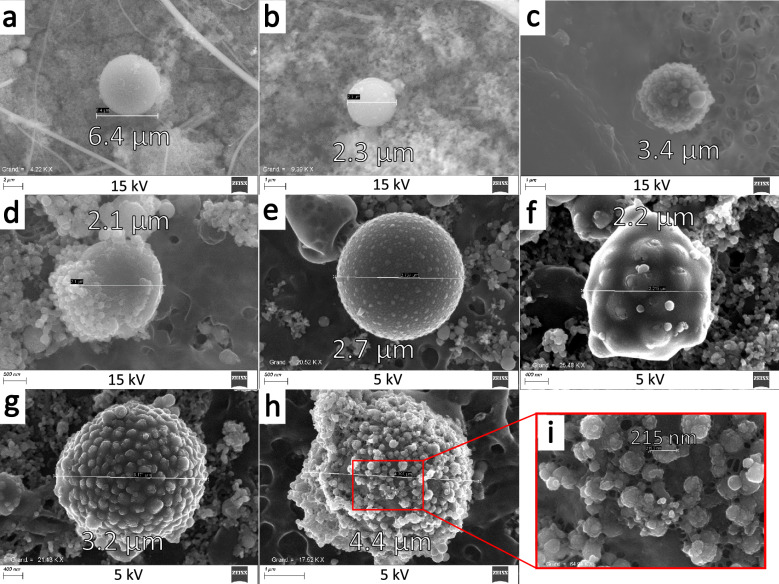
SEM images of synthesised microparticles. Images (**a**–**d**) have been captured at an acceleration voltage of 15 kV and are particles resulting from the test section with a closed reaction chamber. (**a**,**b**) are spherical particles with a smooth surface, (**c**) looks like a agglomeration of submicrometric-sized particles and (**d**) is a smooth surface particle half-covered with submicrometric-sized particles. Images (**e**–**i**) were captured at an acceleration voltage of 5 kV. These particles are resulting from the test section without reaction chamber and do not have a smooth surface. (**e**–**g**) are bumpy particles with a varying intensity and size of bumps. (**h**) is a particle with a distinctive layer stuck on the surface, and (**i**) a zoom on this particle showing with details the surface and the bonds between the submicrometric-sized particles.

The detailed observations of the micrometric-sized particles led to the initial identification of two distinct categories. Firstly, the majority of these particles have a nearly perfect spherical shape, with potential slight depositions on their surfaces, possibly resulting from the collection of other smaller aerosols (Fig. 1a,b). Within this category, the largest particle observed had a diameter of 6.4 $\upmu$m (Fig. 1a). In general, most of the observed micrometric-sized particles have diameters ranging from about 2–3 $\upmu$m. The second category consists of micrometric-sized particles that appear to be aggregates of submicrometric-sized particles fused together (Fig. 1c). It is highly probable that these aggregates consist of micrometric-sized particles trapped beneath a layer of submicrometric-sized particles that remained attached during the water dispersion step of the sample preparation process, as some of them are only partly covered (Fig.  1d). This assumption is further supported by the fact that these particles typically appear larger than the spherical micrometric-sized particles found in the first category.

The efforts to optimize the experiments led to carry out experiments with a less confined reaction chamber, called open test section and using less fragile ceramic parts. The initial confined test section has a reaction volume of roughly 0,25 L (Figure S3a), while the whole volume of the vessel (70 L) can be considered as the reaction chamber for the open test section (Figure S3b). As a result, a new type of micrometric-sized particle variation was observed (Fig.  1e–g). These micrometric-sized particles will be termed “bumpy” particles hereinafter, owing to their distinct surface appearance. While predominantly having a global spherical shape, they possess a diverse range of surface roughness characteristics. In addition, the evolved experimental test section also resulted in the generation of particles closely resembling those depicted in the Fig. 1d, but entirely covered of submicrometric-sized particles (Fig. 1h). Upon closer examination of the surface, it appears that these particles consist of micrometric-sized particles with attached submicrometric-sized particles on the surface, bound through mechanical bonds. These bonds appear to be the result of the solidification of viscous wires (Fig. 1i).

### Global chemical composition of synthesized CsMPs

In a previous study, we provided an analysis of the chemical composition of the particles synthesized via MCCI under an inert gas (N$_2$) atmosphere^26^. The results demonstrated that the synthesized micrometer-sized particles exhibited a composition quite similar to CsMPs found in Fukushima. This conclusion was drawn through an analysis of their energy-dispersive X-ray (EDX) spectra, which revealed Si and O as the major elements, along with the presence of elements such as Na, Mg, Al, K, Ca, Fe, and Cs. It was observed, however, that the Fe and Cs concentrations in the synthesized particles appeared to be much lower than that found in real CsMPs. Additionally, a notable absence of Zn, an important element, was also identified in the synthesized particles. The source of Zn is yet to be determined as the addition of ZnO in a few experiments did not resulted in a detectable amount of Zn in the observed synthesized particles.

In this study, the experiments were conducted using the same prototypic corium and process than our previous study^26^. Some trials were partially conducted under a reactive atmosphere to investigate the potential impact of steam on particle generation and composition, because it is estimated that the atmosphere inside the PCV at the moment of the lower head failure of the concerned reactor was particularly rich in steam. The reactive atmosphere was injected in the test section at the time of collection of the aerosols and the composition consisted of 20 % (partial pressure) steam and 80 % nitrogen. Additionally, further experiments were performed in a pure nitrogen atmosphere to expand the number of samples analyzed and explore the influence of slight variations in the prototypic corium composition.

**Figure 2:**
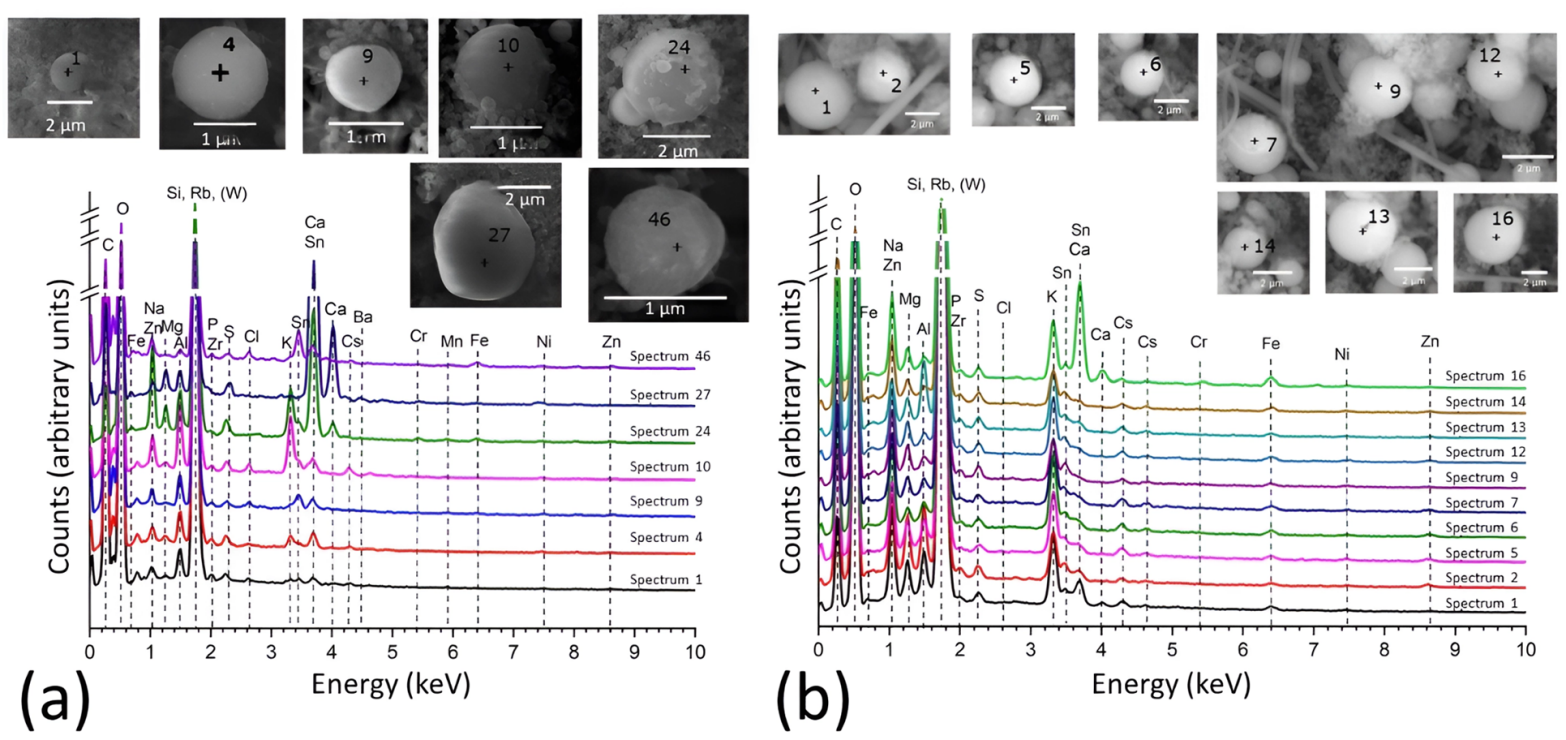
(**a**) SEM-EDX spetra of 7 micrometric-sized particles synthesized over several experiments in dry atmosphere. (**b**) SEM-EDX spetra of 10 particles synthesized in one trial in humid atmosphere.

Seven particles from a prototypic corium of a higher stainless steel content than the previous study and generated in a pure nitrogen atmosphere were analyzed in various experiments (composition in Table S1 and Table S2) (Fig. 2a). As expected, the overall composition appears quite similar to the previous findings, but significant variations in specific element concentrations can be observed among the particles. Apart from Si and O from the matrix, Na, Al, S, and Ca are consistently present in every particle. However, there are observable concentration differences, particularly for Ca. Both Fe and Zn concentrations are very low or undetectable in all particles. The concentrations of Mg, Cl, K, Sn, and Cs vary from one particle to another. In particular, spectra 10 and 24 of the Fig. 2a show higher concentrations of K and Na, suggesting that some factor may have favored the enrichment of alkali elements in these specific cases.

Ten particles from a single trial conducted under a mixed atmosphere were characterized using SEM-EDX (Fig. 2b). The elements found in these particles are generally similar to the experiments done under nitrogen atmosphere, except for Cl that is not detected. The presence of steam seems to have an influence on alkali elements as well, as Na, K, and Cs have been consistently measured in every particle and in significantly higher amounts. Fe was also detected in all particles. With the exception of spectrum 16 of the Fig. 2b, Ca was found to be present in very low concentrations in each particle. It is worth noting that the compositions of these particles might show more homogeneity compared to previous experiments, likely due to the fact that they originate from the same trial.

**Figure 3:**
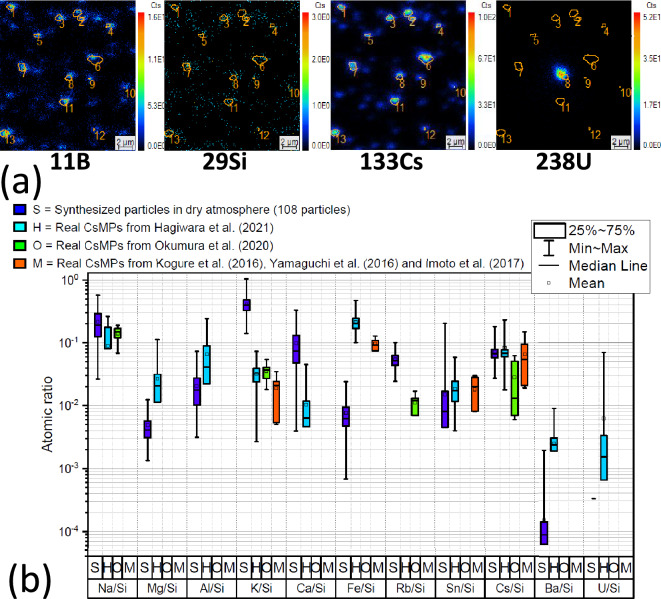
(**a**) SIMS ionic images of several clusters of $^{11}$B, $^{29}$Si, $^{133}$Cs and $^{238}$U identified as micrometric-sized particles. Potential CsMPs were identified using only Si and Cs ionic images. $^{29}$Si has been chosen here due to the saturation of the detector caused by $^{28}$Si. All of the identified particles contains B, and one particle contains U in this image. (**b**) Comparison of the atomic ratio values of Na, Mg, Al, K, Ca, Fe, Rb, Sn, Cs, Ba and U on Si, between 108 synthesized particles in a dry atmosphere and data from real CsMP. Hagiwara et al.^34^ studied 37 particles exhaustively, Okumura et al.^40^ provides alkaline species concentration of 5 particles and Kogure et al.^5^, Yamaguchi et al.^8^ and Imoto et al.^33^ provide the concentration of major elements, all of them are not detailed here (7 particles). It should be noted that studies prior to Okumura et al.^40^ did not identified Na due to overlap with Zn. Ratios without minimum value infer that some particles have a concentration equal to zero for this specific element.

Secondary ion mass spectrometry (SIMS) analyses were conducted on particles generated in a pure nitrogen atmosphere to measure the concentration of the different minor elements and especially detect the presence of B and trace elements such as U. The identification of the particles was made considering Si and Cs clusters as being particles (Fig. 3a). Using relative sensitivity factors, the relative composition of 108 particles was determined^31^. B was measured in significant concentrations, with a median B/Si atomic ratio value of $8\times 10^{-2}$. A specific study^32^ reported that CsMPs contains 1518–6733 mg kg$^{-1}$ of $\mathrm {^{10+11}}$B. For a CsMP composed of 60 % SiO$_2$ by mass, the atomic ratio of B/Si thus varies from $\mathrm {1.4\times 10^{-2}}$ to $\mathrm {6.2\times 10^{-2}}$. It was found that the synthesized particles are slightly richer in B compared to real CsMPs. Among the 108 analyzed synthesized particles, 25 of them contained some U at low concentrations.

The synthesized particles were subjected to a comprehensive analysis of the relative atomic concentrations of Na, Mg, Al, K, Ca, Fe, Rb, Sn, Cs, Ba, and U relatively to Si. Figure 3b illustrate the statistical processing of the atomic concentration ratios for the 108 synthesized particles analyzed, juxtaposed with a parallel statistical analysis of the composition of authentic CsMPs data from the literature^5,8,33,34^. The investigation by Hagiwara et al.^34^ presents the most exhaustive composition analysis over 37 particles, and Fig. 3b illustrates the commonly measured elements. Upon comparing the median concentration values between real CsMPs and synthesized particles, Fig. 3b reveals that Na, Mg, Al, Sn, Cs, and U show a Si relative concentration quite similar, with a difference of less than one order of magnitude. However, for K, Ca, Fe, and Ba, the median concentration diverges more significantly, exceeding one order of magnitude. Focusing specifically on the alkaline species concentration, the synthesized particles show a comparable concentration for Na. The concentration in Cs is almost identical, but marginally lower than that of the real particles from Okumura et al.^40^, given the utilization of composition data from the particle centers for those having a Cs concentration gradient. For K, the concentration in the synthesized particles is higher in the same extent than observed in the previous study. Regarding Rb, the concentration in the synthesized particles is slightly higher; however, it is noteworthy that Rb is introduced as a distinct component in the simulant corium mixture, and its concentration can be readily adjusted. Considering the data from Kogure et al.^5^, Yamaguchi et al., and Imoto et al.^33^, the concentration is consistent for Sn and Cs. Nonetheless, the findings align with the preceding data for K and Fe. It is important to note that these earlier data were collected prior to the confirmation of Na’s presence in CsMPs. Consequently, the Na concentration was initially misinterpreted as Zn additional concentration due to peak overlap in EDX analysis. Overall, Na, Sn, and Cs show highly similar concentrations, while K and Ca concentrations are higher in synthesized particles, and Fe concentration is considerably lower.

### Internal structure of synthesized CsMP

**Figure 4:**
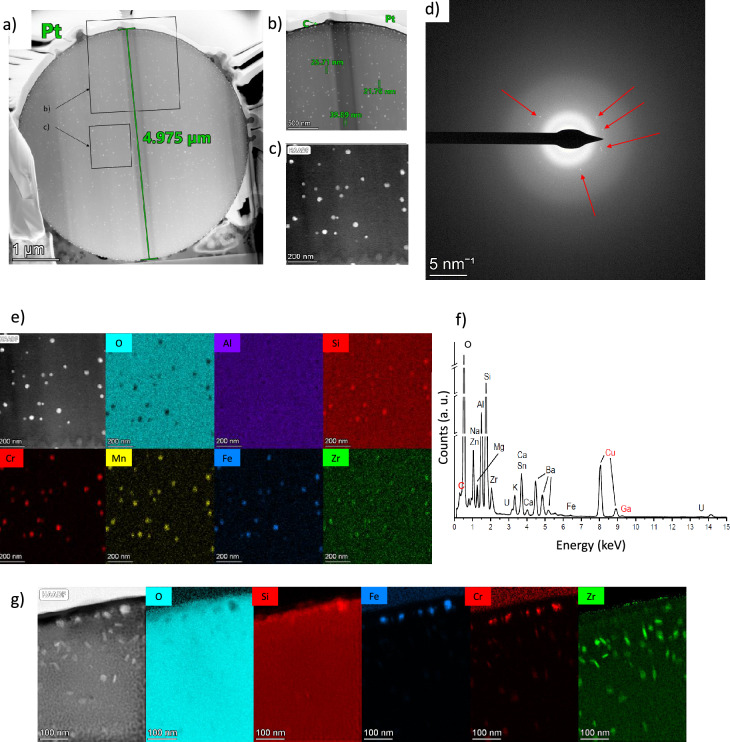
(**a**) STEM dark-field image of the particle OPT-1 showing many crystalline inclusions (bright spots), more visible on (**b**,**c**) enlarged images. (**d**) is diffraction images of the area (**c**), showing a mostly amorphous phase and a few diffraction patters from the crystalline nano-inclusions pointed out by red arrows. (**e**) shows the STEM-EDX elemental maps of the area (**c**) for interest elements constituting the crystalline inclusions of Fe, Cr, Si, Mn and Zr. (**f**) is the EDX spectrum of the same area, knowing that Cu and Ga have been brought by the preparation process or the sample holder, and that C has also mostly been brought by C coating. (**g**) is the elemental map of a smaller area near the top boundary of the particle showing inclusions rich in Zr.

**Figure 5:**
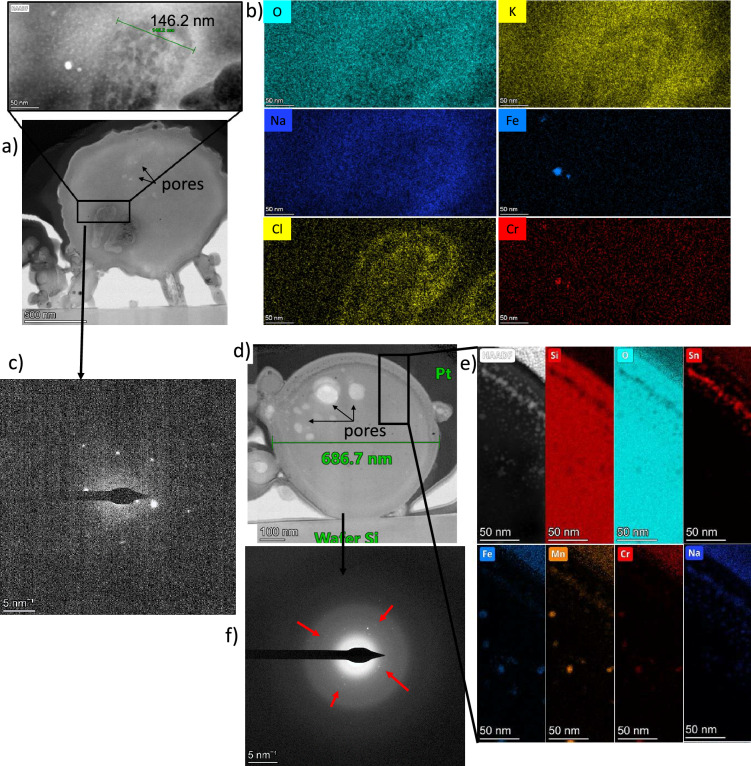
(**a**) TEM image of the particle OPT-2 with a STEM dark-field enlargement of a crystalline inclusion area. (**b**) elemental map of the enlarged area of OPT-2 showing an enrichment in Cl and Na. (**c**) diffraction pattern of the same area showing a crystalline pattern. The detector was saturated rendering a poor contrast. (**d**) TEM image of the particle OPT-3. (**e**) Elemental map of an enlarged area of the particle OPT-3 showing inclusions similar to those observed in particle OPT-1, as well as Sn-rich inclusions near the boundary. (**f**) Diffraction pattern of the particle OPT-3 showing an amorphous matrix and diffraction patterns pointed out by the red arrows.

The internal structure of three synthesized CsMPs have been characterized through the preparation of thin sections using focused ion beam (FIB) techniques. It has been confirmed via diffraction analysis on a transmission electron microscope (TEM) that the silica matrix of the synthesized particles consists of an amorphous phase (Fig. 4d, 5c,f). Nanometer-sized crystalline inclusions and pores have been observed within the particles. The largest particle observed (OPT-1), having a diameter about 5 $\upmu$m, does not contain any pore, but a high concentration of crystalline inclusions is present (Fig. 4a–c). The first type of crystalline inclusions mainly consists of round precipitates, each with a diameter smaller than 40 nm. These precipitates are composed of Fe, Cr, Mn, Si, and Zr and appear to be uniformly distributed throughout the particle (Fig. 4e,f). Another type of crystalline precipitates is distinguishable around the periphery of the particle’s thin section, at depths up to 200–300 nm from the particle’s surface. These inclusions have roughly the same size than the other inclusions but have an elongated projected shape and are predominantly composed of Zr without any visible impoverishment in oxygen content compared to the matrix (Fig. 4g).

The particle OPT-2 has a diameter of 1.4 $\upmu$m and contains pores and precipitates (Fig. 5a and S4a). The porosity is mostly concentrated in the upper region of the thin section, occupying an area of an approximate diameter of 500 nm. The pores display numerous irregular shapes and varying sizes, with their maximum Feret size reaching around 50 nm. This particle also has an amorphous matrix and contains only a small number of nanometer-sized crystalline inclusions such as in OPT-1, but contains two significant crystalline inclusions occupying an area of approximately 400 $\times$ 400 nm (Fig. 5c).

The chemical composition analysis of these particles has proven challenging due to uncertainties arising from the use of a focused beam in scanning transmission electron microscopy (STEM) configuration during EDX measurements. This is known to alter the composition and structure of the materials being analyzed, particularly affecting alkali species, which have a propensity to migrate within the matrix. Nevertheless, the elemental map obtained from the upper part of the left precipitate suggests that this specific inclusion is abundant in chlorine (Cl) and deficient in alkali species, especially potassium (K) and sodium (Na) (Fig. 5b).

A third particle, designated as OPT-3, has been subjected to internal structure analysis. Its diameter is approximately 700 nm, which is smaller than the typical CsMPs size. Similar to the previous particle, OPT-3 also contains pores, with the largest pore having a diameter of about 100 nm (Fig. 5d and S4b). These pores are predominantly located in the upper left part of the particle. Additionally, OPT-3 contains spherical precipitates that are rich in Fe, Cr, Mn, Si, and Zr, similar to what was observed in OPT-1. Furthermore, some other nanometer-sized inclusions, enriched almost exclusively in Sn, have been identified near the surface of the particle (Fig. 5e,f). It is worth noting that the thin section of this specific particle was the thinnest among the three particles, measuring approximately 80 nm, while the others were 117 nm and 153 nm thick for OPT-2 and OPT-1 respectively (Figure S5a–c). This lower thickness probably increased this thin section structural modifications under the electron beam during the analysis process (Figure S6).

During the thin section preparation process, several submicrometer-sized particles were inadvertently trapped in the platinum (Pt) material. Due to the likelihood that the sections were not made near the equator of these particles, the observations obtained may not be fully representative from one particle to another. However, it has been observed that the majority of these trapped particles contain at least one nanometer-sized precipitate, similar in nature to those observed in OPT-1 and OPT-3.

## Discussion

It has been reported that aerosols resulting from MCCI typically have a diameter exceeding 0.4 $\upmu$m, with a predominant size range of several micrometers^35^. While the largest particles may exceed 10 $\upmu$m, it is suggested that these particles primarily arise from mechanical generation processes, such as matter entrainment, rather than nucleation and condensation, as explored in our study^36,37^. Type-A CsMP have always been observed having a diameter smaller than 10 $\upmu$m, which agrees with a formation by MCCI regarding their size distribution. A specific investigation has reported the existence of type-A CsMPs with a diameter less than 0.5 $\upmu$m^38^, implying that the isolation of CsMPs based on the amount of Cs they contain (i.e. radioactivity) remains the most reliable method for detecting the most harmful particles in terms of dose, as well as the largest particles. Given that the size distribution of the particles synthesized in this study is slightly smaller than the typical size distribution for MCCI aerosols, it is hypothesized that the rapid cooling induced by gas injection forced convection in the test section, coupled with a high prevalence of nucleation sites caused by the confined reaction volume, favored the creation of numerous smaller particles rather than promoting growth through continuous condensation, leading to the formation of larger particles.

**Figure 6:**
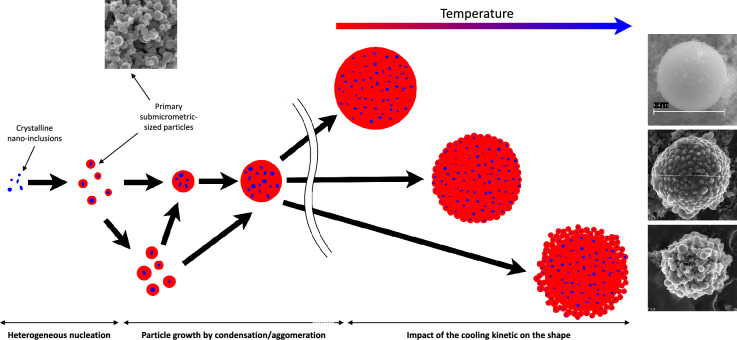
Suggested mechanism of generation for the synthesised particles in relation to the observations on the particles size distribution and their internal structure.

The prevalence of the submicrometric-sized particles, together with the observation of the bumpy micrometric-sized particles has been beneficial in the understanding of the generation process. The observation of the inside of the particles revealed the existence of nanoinclusions such as inside numerous type-A CsMPs^5,23,39^. It also seems that among the submicrometric-sized particles that were unintentionally included in the thin sections, most of them contain at least a nanometric sized inclusion. It is therefore thought that these nanometric inclusions might serve as nucleation sites for silicon rich aerosols generated by the MCCI to condense and create silica particles (Fig. 6). In the case of the experiments, many nucleation sites are available in a really confined volume, and the forced convection enhances this phenomenon by renewing the atmosphere above the corium. This favours the nucleation of many new particles by the condensable gases rich in Si. Particles with a short residence time will be extracted as submicrometric-sized particles, those with a longer residence time might grow by additional condensation and more likely coagulate with other submicrometric-sized particles to form micrometric particles. The conditions inside the containment at 1F were significantly different, in terms of volume, gas velocity and temperature gradient. It is supposed that nucleation sites (i.e. nanoparticles) were sparsely available, favouring a growth of microparticles by condensation mostly. It is worth noting that most SEM images of real CsMPs in the literature were captured at an acceleration voltage of 20 kV and with a back-scattered electron detector, to proceed to the chemical analysis in the meantime. The few low voltage high-resolution images captured of spherical type-A CsMPs tend to show a not so regular surface^8^.

In the evolution of the test section design, particles are cooled down much faster due to the open reaction chamber that provides less thermal confinement into the outlet of the vessel (Figure S3). It is supposed that this is the reason for the existence of bumpy particles. The micrometric-sized particles are probably the result of the coagulation of submicrometric-sized particles at a viscous state, nucleated on the nanometric size mixture of stainless steel elements and zirconium inclusions. The bumpy particles are likely to be witnesses of this coagulation process, by showing the progressing coagulation, interrupted by a fast cooling during the extraction of the aerosols from the vessel. Experimentally, the aerosols are pushed out by a gas flow that forces their cooling by a high velocity rising, compared to what could have occurred in the containment of 1F. This forced cooling is enhanced during the experiments carried out with the open test section and increase the chance of generating “bumpy” particles.

The chemical composition of the nano-inclusions is not as diverse and entirely identical to the observed inclusions in real CsMPs. In the actual accident scenario, nanoparticle sources have a much greater diversity than in our experimental setup. For instance, (U,Pu) oxides are likely the result of the mechanical deterioration of exposed fuel rods, Fe oxides might arise from the mechanical removal of the stainless steel oxide layer, and fission products inclusions may originate from the release of fuel-derived materials. However, the particle OPT-2 contains inclusions rich in Na and Cl (that could be considered as a sodium chloride precipitate), which is really similar to a precipitate of cesium chloride (by extension of alkaline properties) that has been observed in a real CsMP^23^. In the same study, the authors also identify a zinc chloride inclusion that may have been misidentified and could, in reality, be a sodium chloride precipitate, given that Na has been confirmed to exist in CsMPs later and was misidentified as Zn initially^40^. Furthermore, in another study, a particle contain an inclusion made of Fe and Cr, resembling those observed in synthesized particles and another particle an inclusion rich in Cl^15^.

The absence of Ni, a major constituent of stainless steel, in the nanoinclusions might seem surprising. The stainless steel (SS) utilized in these experiments is SS301, chosen due to a supply issue with SS304. Despite this substitution, the composition of SS301 closely resembles that of SS304, containing up to 2wt% of Mn and an equal amount of Si, the latter being slightly higher than in SS304. Mn and Si rank as the fourth most abundant elements in this alloy, following Fe, Cr, and Ni. Consequently, the presence of Mn and Si in these inclusions is unsurprising. Regarding Ni, studies on stainless steel welding have revealed that resulting fumes contain approximately 10 times less Ni than Fe or Cr, despite being only 5 to 6 times less concentrated than Fe and 2 times less concentrated than Cr^41^. Furthermore, SS301 contains a slightly lower amount of Ni in its composition compared to SS304.

The chemical analysis of the synthesized particles reveals differences, especially in the case of Ca, when compared to real CsMPs. Over the years of characterization, it has been observed that the composition of CsMPs may vary from one to another^4^. Ca has been detected in various particles from different locations or substrates, and it has been characterized using multiple techniques^34,42,43^. In this study, experiments conducted in a humid atmosphere appear to generate particles with a low concentration of Ca, indicating that the atmosphere could play a crucial role in influencing the particle composition for certain elements.

SIMS analysis indicates a similarity in Cs concentration between the synthesized particles and the CsMPs, in contrast to observations from SEM-EDX analyses on synthesized particles. This discrepancy may be attributed to the particle identification process. Real CsMPs are isolated from samples, such as soil, utilizing an imaging plate to pinpoint highly radioactive spots. Consequently, the prioritized identification of the most radioactive CsMPs occurs, considering that Cs concentration can vary by approximately one order of magnitude from one particle to another, as reported by a previous study^40^. In the SIMS analyses conducted for this study, particles were also identified by instructing the device to locate clusters rich in Si and Cs. Conversely, SEM-EDX relied solely on morphological features observed in images for particle identification, and the majority had significantly lower Cs concentrations.

It is suggested that the solubility of alkali species might have influenced their concentration for humid atmosphere experiments. In the presence of a highly humid atmosphere, Cs mainly forms CsOH, a highly reactive species^19^. The other alkali species tends to behave in the same way. This highly reactive chemical forms could have enhance the inclusions of alkali species into the particles. Thermodynamic calculations did not show a significative influence of the atmosphere in the concentration of these specific elements as well as Fe and Ca. However, it has been noticed that the data from the Termodynamics of Advanced Fuels-International Database (version 13) might not depict all the real interactions that could occur.

These experiments represent a promising insight into understanding the potential of MCCI to easily generate particles closely resembling type-A CsMPs in terms of shape, size, structure and chemical composition. An exact synthesis of type-A CsMPs is hard due to their relative composition variation and due to the myriads of reaction conditions that occur simultaneously in a failed reactor, considering thermal and pressure gradients, gradients in reactive materials compositions, large compositional variations at different locations and only partial mixing of reactive fluids and with rapid variations over time. Regarding the chemical composition, refining certain parameters could strengthen our conclusions, particularly if more controlled experiments could be conducted in a humid atmosphere and if Zn origin could be identified. Some specific characteristics of the experimental setup are supposed to influence the particle formation, especially their size and shape. An upscaled experiment could lead to a slightly different observation due to a different aerodynamic condition and reaction volume. It is suggested that the particle result from condensation on nano-particles, then growth and agglomeration to form micrometric size particles. Further characterizations of environmental samples from the Fukushima area could concentrate on determining the size distribution of CsMPs compared to experimentally obtained particles.

These findings have shown that an experimental set up designed to simulate some aspects of MCCI in a laboratory has produced Cs-enriched silica microparticles which are in many aspects similar to CsMP observed in Fukushima. Although detecting and identifying this kind of particles during an ongoing accident pose inherent challenges, their recognition could serve as an early indication that MCCI has initiated, prompting a targeted response from crisis management centers. More research is necessary to confirm that the CsMP observed can be confirmed as an indicator of MCCI.

Moreover, considering CsMPs in severe accident codes could prove beneficial for accurately assessing the source term released into the environment. The Fukushima Daiichi incident has shown the substantial contribution of CsMPs to radioactive pollution.

Subsequently, the scientific community may seek to evaluate the impact of particulate-trapped Cs in comparison to ionic Cs, with regard to both the delivered dose to individuals and the transfer of radioactivity to the environment. In the context of designing future reactors, particularly in determining concrete composition, a critical decision must be made regarding whether CsMPs should be promoted or strictly avoided in the event of an inevitable release of radionuclides following the initiation of MCCI.

## Methods

### Experimental setup

The experimental setup comprises a furnace serving as a chemical reactor to induce MCCI and a sampling line for collecting and analyzing aerosols. The furnace, a water-cooled stainless steel vessel with a volume of approximately 70 L, contains the test section and a water-cooled inductor with four turns. A 50 kW electric generator (operating at about 110 kHz) supplies power to the inductor, establishing electromagnetic coupling with a conductive component (susceptor) to generate heat (Figures S7 and S8).

The test section is composed of an yttria fully stabilized zirconia (Y-FSZ) crucible, a tungsten cylinder (susceptor) for electromagnetic coupling, and a thermal shield made of porous graphite. To address the reactivity of tungsten and graphite with a humid atmosphere at high temperatures, a test section with a reaction chamber made of Y-FSZ has been developed to prevent the atmosphere from reaching the tungsten and graphite. This configuration, providing only moderate airtightness and limited mechanical resistance to thermal gradients, could not satisfy the requirements. An alternative version without the Y-FSZ reaction chamber has been developed, the entire vessel being the reaction chamber, only allows experiments in a dry atmosphere (Figure S3).

The sampling line extracts aerosols from the top of the vessel, dilutes the flux with nitrogen (at a 1:6 ratio) to reduce particle concentration, and splits the diluted flux into three streams. One stream passes through a particle counter (Pegasor®PPS-M), another through a HEPA filter (0.2 $\upmu$m pores) for particle collection, and the third through a low-pressure impactor (Dekati DLPI+) to measure the inertial size distribution of particles. The sampling line is heated to over 100 $^\circ$C to prevent water condensation.

Temperature measurements employ two bichromatic video pyrometers, one focused on the corium (from the top of the vessel) and another on the crucible or reaction chamber (depending on the test section version). However, measurements above 1700–1800 $^\circ$C are deemed unreliable due to the substantial aerosol generation perturbating the optical path. Calibration tests with an empty crucible were conducted before real experiments to estimate crucible temperature based on power injection.

### Test protocol

The experiments involve the interaction between sub-oxidized corium and basaltic concrete resembling that of 1F. Corium compositions are determined using the results of the BSAF project^44^. The corium is prototypic as it utilizes depleted uranium oxide and does not incorporate any other radioactive isotopes (including PF), but only as stable isotopes or excludes them if non-existent in their stable form (Tables S1 and S2). This composition, rich in UO$_{2}$ and sub-oxidized Zr is typical of an early accidental phase.

The concrete has been made with materials from Japan close to the originals constituting 1F’s concrete. The composition has been analyzed using inductively coupled plasma atomic emission spectroscopy (ICP-AES) and shows that the concrete is made from 58.7wt% of SiO$_{2}$, 9.9% of CaO, 8.4% of Al$_{2}$O$_{3}$, 5.8% of Fe$_{2}$O$_{3}$, 2.4 of Na$_{2}$O, 1.5 of K$_{2}$O and 1.3 of MgO. The concrete also contains many other minor or traces elements (as oxides) such as Ti, Pb, S, Ba, etc. The concentration of water and carbonates in the concrete could not be measured. Basaltic concretes usually have a low concentration in carbonates. The water content is estimated to be around 6–8wt%.

The mixture is loaded into the crucible as two configurations: a homogeneous mixture with all elements mixed as powders and a heterogeneous mixture where the sub-oxidized corium and concrete are vertically separated by a stainless steel sheet (no visible influence on the aerosols). After installing the test section in the vessel and loading the mixture, the vessel is hermetically sealed and subjected to medium to high vacuum. It is then filled with nitrogen to atmospheric pressure. The heating process starts slowly to limit powder projection caused by rapid gas expansion (reaching 1200–1300 $^\circ$C in about 30 min). Subsequently, the temperature is abruptly increased to minimize the interaction time until the target MCCI temperature of 2000–2200 $^\circ$C is reached. Aerosol collection begins after reaching the target temperature by opening the sampling line and injecting a gas flow from the bottom of the test section to carry the aerosol to the top and prevent pressure decrease in the vessel. The injected gas flow may consist entirely of nitrogen or be a humid gas composed of mixed nitrogen and steam.

### SEM-EDX samples preparation and observations

Particles under study are collected on a HEPA filter. A portion of less than 1 cm$^{2}$ is taken from the filter with the deposit, transferred into 4 mL of distilled water, and vigorously agitated for about 1 min to disperse particles and dissolve soluble aerosols. The suspension is filtered through a 0.2 $\upmu$m pore membrane, and the membrane is let to dry for a minimum of 24 h at room temperature. Subsequently, the membrane with the low-solubility particle deposit is transferred onto a stainless steel SEM stub, affixed with double-sided carbon tape, coated with carbon, and supplemented with a piece of aluminum tape to enhance electrical discharge.

Analyses have been conducted on a Zeiss Sigma 300 VP SEM. General observations use an Everhart–Thornley-type detector at 15 or 20 kV, simultaneously with EDX analyses. High-resolution images are captured at 5 kV using a secondary electron InLens detector.

### SIMS samples preparation and observations

SIMS samples are prepared identically to SEM samples until the coating step. Particles are then suctioned using a vacuum system onto a carbon stub previously coated with an adherence polymer.

Analyses have been carried out on a Cameca-Ametek IMS 1300HR$^{3}$ LG-SIMS using an oxygen primary beam.

The intensity of the signal measured with SIMS is not directly related to the concentration of the isotopes in the sample. It is necessary to consider the ionization potential of the element of interest, as well as the matrix from which it is extracted. The best way to perform such kind of measurement is to use a reference material with a similar known composition. Unfortunately, no such kind of material was available, or any real CsMP. In consequence, the relative sensitivity factor (RSF) method has been used. Based on measurements carried out on a similar matrix (SiO$_{2}$) by other researchers, the RSF of each isotope can be determined. Considering the element X that has been measured through its isotope $\mathrm {^{N}}$X, its relative atomic concentration to Si can be calculated as follow :

1 $$\begin{aligned} \frac{X}{Si}=\frac{i~_{^{N}X}}{i~_{^{28}Si}} \times \frac{a~_{^{28}Si}}{a~_{^{N}X}} \times \frac{RSF_{X}}{\rho _{Si}} \end{aligned}$$

with *i* being the ionization potential, *a* the abundance of the concerned isotope and $\rho$ the matrix atomic density.

### TEM-EDX samples preparation and observations

Thin sections have been prepared using an FEI Helios 600 NanoLab. Particles are laid on a silicon wafer, affixed with silver coating. In the FIB-SEM, electronic and ionic Pt deposition secures the region of interest, and micro-machining is achieved by a Ga+ source starting from 30 kV.

Thin sections are then transferred onto a Cu holder for TEM observations, conducted using an FEI Talos F200S at 200 kV.

### Supplementary Information

Supplementary Information.

## Data Availability

The data will be made available upon request, and interested parties may obtain it by contacting the corresponding author (Christophe Journeau—christophe.journeau@cea.fr).
