# Supplementary material for: Simulant molten core–concrete interaction experiments in view of understanding Fukushima Daiichi Nuclear Power Station Cs-bearing particles generation mechanism
Source: Sci Rep. 2024 Mar 19;14:6611. doi: 10.1038/s41598-024-56972-9 (PMC11344152; doi:10.1038/s41598-024-56972-9)
Supplement: Supplementary file 1 — Supplementary Information. [file 41598_2024_56972_MOESM1_ESM.pdf]

Supplementary information

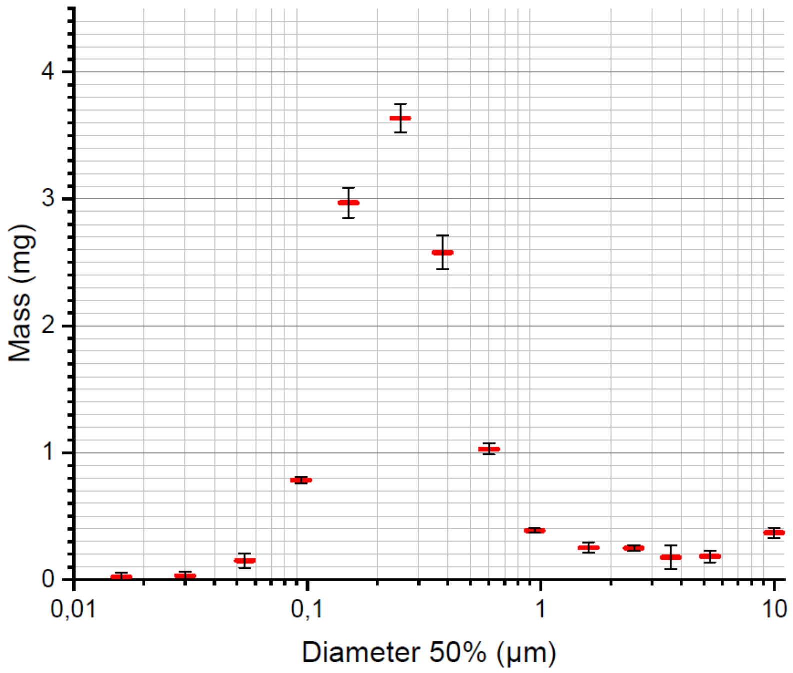

Figure S1. Size distribution measurement. X-axis is the median aerodynamic diameter of the particles.

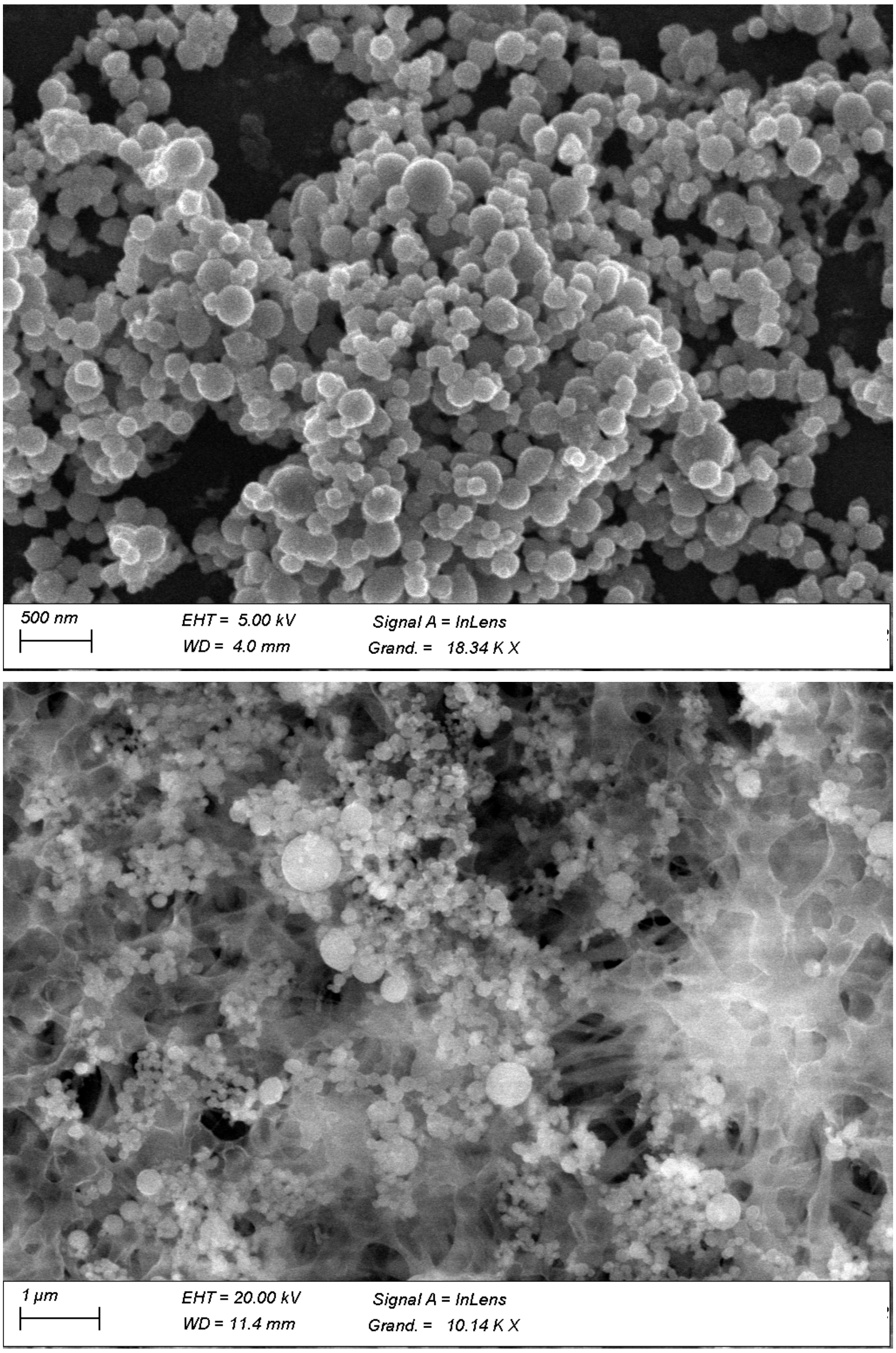

Figure S2. SEM images used for particle counting with the CELLPOSE algorithm.

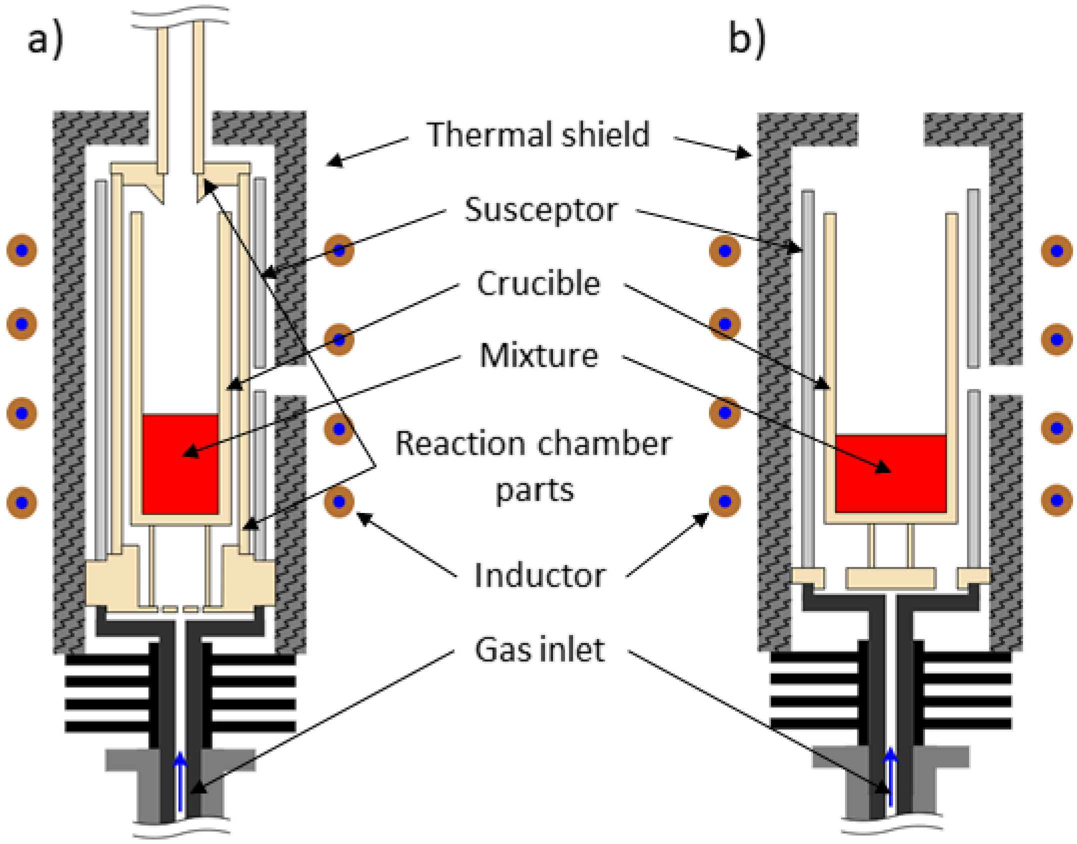

Figure S3. Test section a) with the reaction chamber and b) open.

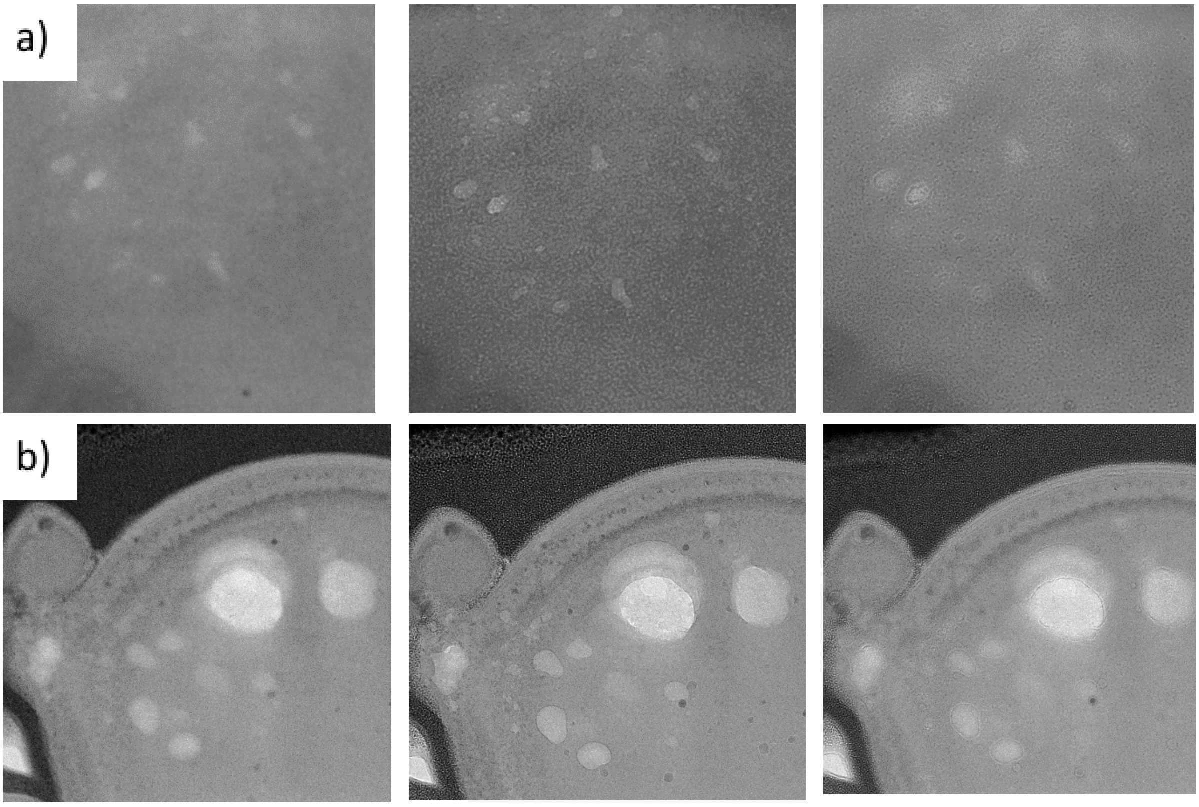

Figure S4. Focus variation for the confirmation of pore presence in particles OPT-2 (a) and particle OPT-3 (b).

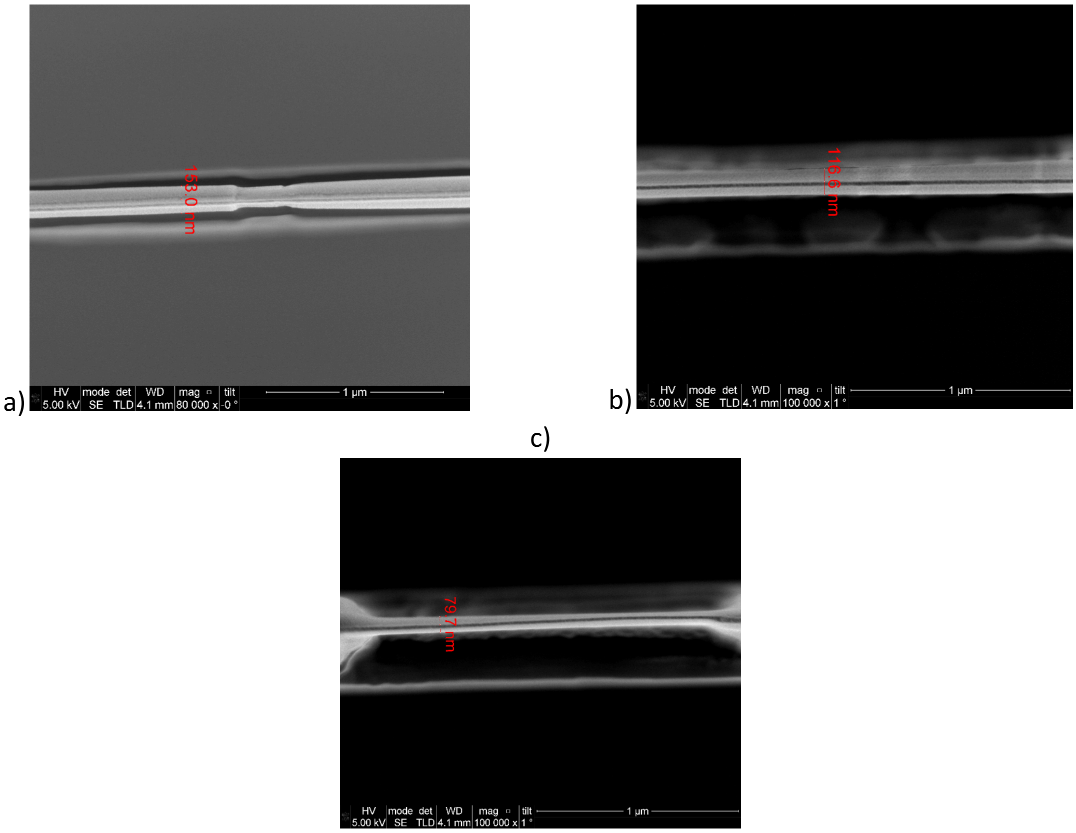

Figure S5. Thin sections thickness measurements of respectively, 153 nm for OPT-1 (a), 117 nm for OPT-2 (b) and 80 nm for OPT-3 (c).

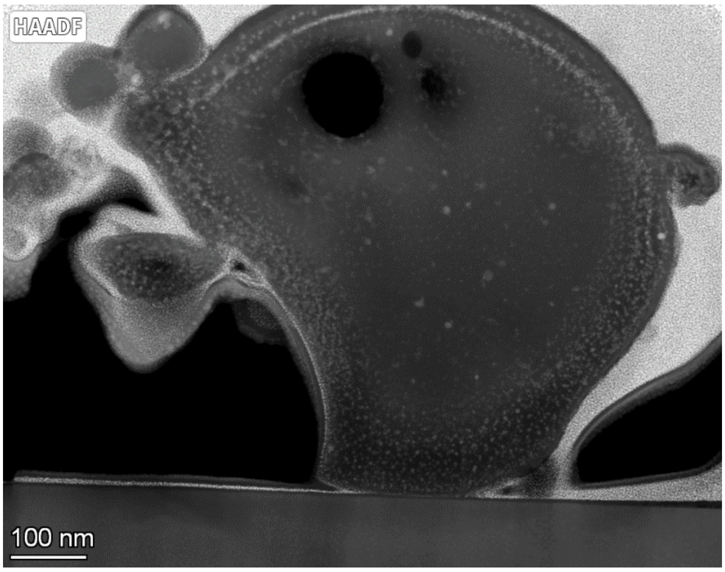

Figure S6. HAADF image of the shape modification underwent by the particle OPT-3 after several minutes under the electron beam.

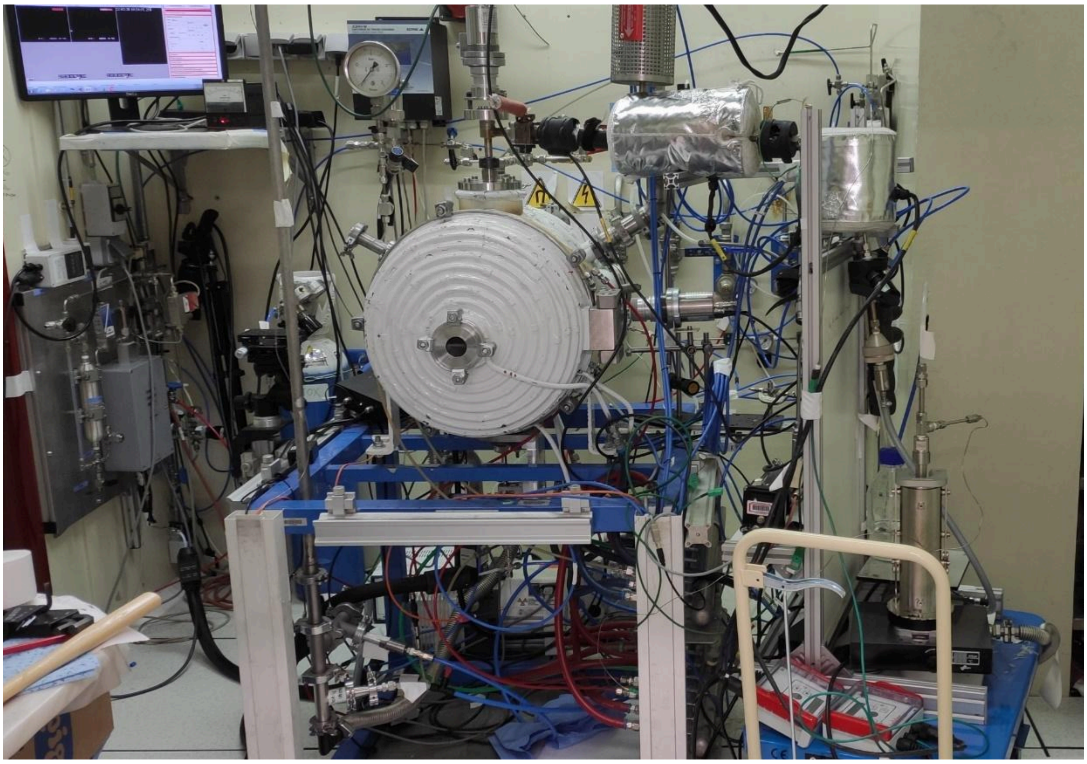

Figure S7. VITI facility in aerosol configuration.

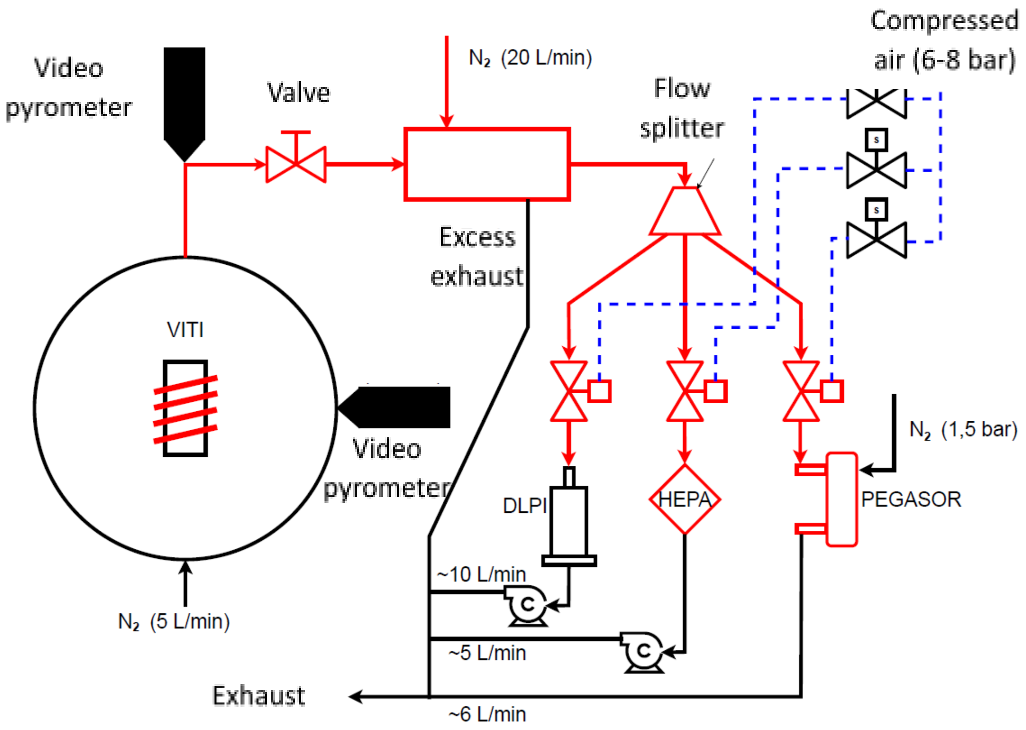

Figure S8. Facility schematic view.

Table S1. Corium composition.

Species (wt%)	Initial corium	Stainless steel rich-corium
UO_2	36.72	34.75
Zircaloy 2	36.72	34.75
ZrO_2	15.88	14.75
Stainless steel	5.96	10.00
Fe_2O_3	2.38	3.00
Cr_2O_3	0.60	1.00
B_4C	0.99	1.00
SnO_2	0.13	-
Cs_2SiO_3	0.04	0.10
ZnO	0.08	0.10
RbCl	0.01	0.05
Fission products	0.48	0.50

Table S2. Fission products mix composition.

Species (wt%)	
Nd2O3	23,00
MoO2	18,30
CsOH.H20	15,60
BaO	9,40
La2O3	6,90
PdO	6,50
Pr2O3	6,50
Sm2O3	3,20
SrO	4,70
Y2O3	3,10
TeO2	2,90
